# P-959. Evaluation of Online Medical Education to Improve Pre-Exposure Prophylaxis (PrEP) Knowledge and Competency among Providers

**DOI:** 10.1093/ofid/ofae631.1149

**Published:** 2025-01-29

**Authors:** Samantha A Devlin, Eleanor E Friedman, Nikki Kasal, Kelly Ducheny, Amy K Johnson, Geoffroy Liegeon, Jessica Ridgway

**Affiliations:** University of Chicago, Chicago, Illinois; University of Chicago, Chicago, Illinois; University of Chicago Pritzker School of Medicine, Chicago, Illinois; Howard Brown Health, Chicago, Illinois; Ann & Robert H. Lurie Children's Hospital of Chicago, Chicago, Illinois; University of Chicago, Chicago, Illinois; University of Chicago Medicine, Chicago, Illinois

## Abstract

**Background:**

Pre-exposure prophylaxis (PrEP) use for HIV prevention remains low among Black women in the U.S. compared to their need. Barriers to uptake include provider discomfort prescribing PrEP to this group. We sought to evaluate the impact of medical education on providers’ knowledge and attitudes toward PrEP among Black women.

**Methods:**

Providers at two community health centers in Florida and Illinois participated in a live, online PrEP training and completed an online pre- and post-test. The training lasted 45-60 minutes and included didactic and interactive content to equip providers with the clinical knowledge for prescribing PrEP (e.g., indications/side effects, PrEP during pregnancy/breast-feeding, maintenance of therapy) while building their competency discussing PrEP with Black women (e.g., culturally-competent and “health-based” rather than “risk-based” scripts). The test evaluated objective knowledge of PrEP, attitudes toward PrEP and discussing PrEP with/prescribing PrEP to Black women, and likelihood of prescribing PrEP in the future. Data were analyzed descriptively.

**Results:**

Nineteen providers completed the pre-test and fifteen providers completed the post-test. Most were physicians or nurse practitioners (68%, 13/19 and 80%, 12/15, respectively) and female (63%, 12/19 and 60%, 9/15, respectively). In the pre-test, 32% (6/19) of participants thought that pregnant women should not use PrEP; 100% (15/15) of post-test participants understood that PrEP was safe during pregnancy. In terms of *discussing* PrEP with Black women, originally 53% (10/19) of participants rated their comfort level as “Poor” or “Fair”, which fell to 13% (2/15) after the training. In the pre-test, 47% (9/19) of participants rated their comfort level in *prescribing* PrEP to Black women as “Poor” or “Fair”, which decreased to 20% (3/15) in the post-test. When asked how likely they were to prescribe PrEP to a Black woman within the next 6 months, 42% (8/19) of participants stated “Likely or “Very Likely” which increased to 80% (12/15) after the training.

**Conclusion:**

Knowledge of PrEP, comfort discussing PrEP with Black women, and intention to prescribe PrEP all improved post-education. Additional data is needed to evaluate the long-term impact of the training on PrEP uptake among Black women.

**Disclosures:**

**Jessica Ridgway, MD**, Gilead Sciences: Expert Testimony

